# Human rights as a framework for eliminating female genital schistosomiasis

**DOI:** 10.1371/journal.pntd.0010165

**Published:** 2022-03-03

**Authors:** Caitlin R. Williams, Maximillian Seunik, Benjamin Mason Meier

**Affiliations:** 1 Department of Maternal and Child Health, Gillings School of Global Public Health, University of North Carolina at Chapel Hill, Chapel Hill, North Carolina, United States of America; 2 Department of Mother and Child Health, Institute for Clinical Effectiveness and Health Policy (IECS-Argentina), Buenos Aires, Argentina; 3 Young Diplomats of Canada, Toronto, Ontario, Canada; 4 Department of Public Policy, Department of Health Policy & Management, University of North Carolina at Chapel Hill, Chapel Hill, North Carolina, United States of America; Weill Cornell Medical College, UNITED STATES

## Abstract

Female genital schistosomiasis (FGS) affects tens of millions of women and girls in sub-Saharan Africa, yet this inequitable threat is often overlooked by advocates in both the neglected tropical disease (NTD) and sexual and reproductive health and rights (SRHR) communities. FGS causes both acute infection and long-term sexual and reproductive health harm to marginalized women and girls, with gender, poverty, and rurality combining to invisibilize the disease. Human rights and gender imperatives can help to galvanize efforts to control and eliminate FGS, as they have for other NTDs. Specifically, international human rights obligations can frame state efforts to address FGS across healthcare settings, upstream social determinants of health, scientific research, and policy implementation. This article analyzes human rights–based approaches to FGS control and elimination efforts, outlining several areas for forward-looking reforms to health policy, programing, and practice. Building from the lessons learned in applying human rights–based approaches to advance progress on other NTDs, this analysis seeks to provide the NTD community with shared understanding around international legal obligations to engage SRHR advocates and draw heightened attention to FGS. Such human rights–based approaches to FGS control and elimination can help to reduce stigma and improve care for the millions of women and girls currently affected by this preventable disease.

## Author summary

Female genital schistosomiasis (FGS) affects over 56 million women and girls in sub-Saharan Africa alone, yet this inequitable threat is largely ignored by global health advocates, program managers, and policymakers. The lack of international attention undermines efforts to eliminate schistosomiasis globally. International human rights law can help to reverse this neglect and shape the role of the international community in supporting elimination of FGS as a public health problem, as has been done for other neglected tropical diseases (NTDs). This analysis provides the NTD community with shared language around international legal obligations to engage sexual and reproductive health and rights (SRHR) advocates and draw attention to this often overlooked condition. Working together, these two communities can reduce stigma and improve the health and well-being of the millions of marginalized women and girls affected by this preventable disease.

In January 2021, the World Health Organization (WHO) formally launched a new roadmap to guide neglected tropical disease (NTD) elimination and control over the coming decade (“NTD roadmap”) [1]. Setting overarching goals and targets and outlining national strategies, this NTD roadmap will guide future technical efforts to address NTDs in meeting the Sustainable Development Goals. The NTD roadmap seeks to incorporate human rights and gender imperatives that have been increasingly mainstreamed in global health governance [2]. This article illustrates how human rights and gender analyses can enrich NTD policy, programming, and practice to achieve global targets around female genital achistosomiasis (FGS) control and elimination as a public health problem [3].

## Female genital schistosomiasis: A long-overlooked sexual and reproductive health and rights concern

Schistosomiasis is acquired upon contact with water infested with larval schistosomes, typically during agricultural and domestic activities. There are two presentations of the disease—intestinal schistosomiasis and urogenital schistosomiasis—which are caused by different species of *Schistosoma*. Of these, *Schistosoma mansoni*, *Schistosoma japonicum*, *Schistosoma mekongi*, and *Schistosoma intercalatum* primarily infect the gastrointestinal tract, while *Schistosoma haematobium* causes urogenital infections. This paper is focused on the latter presentation, which can cause FGS when left untreated or when lesions persist beyond successful chemotherapy.

Despite affecting millions worldwide, FGS is often neglected in global health, although this is slowly beginning to change under initiatives designed to raise FGS awareness. WHO released a pocket atlas on FGS for health professionals in 2015 [4], FGS has been highlighted at international scientific meetings [5], and there are ongoing calls for NTD researchers to focus on FGS [6,7]. The new WHO NTD roadmap, which elevates schistosomiasis as a candidate for elimination as a public health problem (defined as <1% proportion of heavy intensity schistosomiasis infections, as measured by microscopy, filtration for *S*. *haematobium*), specifically names FGS as a discrete issue that requires targeted attention within broader schistosomiasis control and elimination efforts [1].

Current efforts to achieve schistosomiasis control and elimination as a public health problem combine a variety of strategies, including mass drug administration (MDA) campaigns, wide-scale field surveys for mapping and monitoring human transmission, and xenomonitoring and snail control to disrupt chains of snail-to-human transmission [8]. While the WHO 2020 target around schistosomiasis focused solely on coverage of school-age children with preventative chemotherapy through MDA, the new NTD roadmap lays out an integrated set of core strategic interventions that also encompass access to clean water, vector control, and veterinary public health [1]. In addition, the NTD roadmap calls for explicit attention to “diseases for which women and children are disproportionately at risk or for which there are particular manifestations in women” such as FGS [1, p. 57]. However, the roadmap falls short of providing a detailed human rights and gender analysis of FGS and specific recommendations for addressing identified challenges.

The true prevalence of FGS is unknown, affecting at least 56 million women and girls in sub-Saharan Africa alone [9] (while we use the terms “female,” “women,” and “girls” for consistency with the field, it is worth noting that FGS can also affect trans*, intersex, gender nonbinary, and gender nonconforming individuals with vulvas, vaginas, cervixes, uteruses, oviducts, and/or ovaries). There remains a paucity of research on FGS and male genital schistosomiasis (MGS) as compared to other clinical schistosomiasis presentations, impeding diagnosis, treatment, and elimination [10]. Focusing on the health of women and girls, FGS is a critical sexual and reproductive health and rights (SRHR) concern. Lesions in the vulvar, vaginal, and cervical tissue can be mistaken for sexually transmitted infections [11,12]. Egg deposition in the ovaries, uterine tubes, and uterus can cause lasting damage to the reproductive tract [7]. FGS contributes to a wide range of adverse pregnancy-related outcomes and has been associated with prevalent HIV infection [7,10,13–17].

Gender is deeply implicated in FGS incidence and neglect [18]. Gender norms that situate women and girls as the primary collectors of water, workers in the household, and caregivers in the family structure gendered exposures to infested water [19–21]. Women seeking health services often present with nonspecific symptoms, like painful urination or infertility, and consequently face high rates of misdiagnosis, with inadequate laboratory diagnostics undermining appropriate medical treatment [22]. Such challenges for women and girls, compounded by stigma surrounding sexual and reproductive health, can result in neglect of prevention, denial of treatment, poor quality care, and abuse [23].

Despite the primacy of gender in mediating women’s and girls’ enjoyment of the right to health in the context of FGS, global efforts to address schistosomiasis have often lacked a robust gender and rights analysis. The World Health Assembly’s 2001 endorsement of preventative chemotherapy through MDA focused primarily on school-age children. Although the Assembly resolution included women as a priority group and WHO recommended the inclusion of pregnant and lactating women [24], policy implementation lagged until a 2018 WHO evidence summary spurred the United States Food and Drug Administration to approve praziquantel during pregnancy and lactation [25]. Even with this technical guidance, MDA interventions at the local level still sometimes overlook pregnant women [26].

Gender, poverty, education status, and rurality render those affected by FGS invisible, including within the SRHR and NTD communities, although this is beginning to change. FGS presents inequitable harm to marginalized women and girls, yet this endemic threat remains understudied and underfunded relative to other conditions with similar prevalence. By explicitly countering the power differentials that fuel these dynamics, human rights offer a powerful framing for policy efforts to overcome the neglect of FGS.

## The utility of human rights in framing FGS efforts across policy, programming, and practice

Human rights obligations, grounded in a recognition of the equal dignity of all persons, provide a path to realize the universal freedoms and entitlements that underlie health. Evolving under international law through the United Nations (UN), human rights encompass both a right to health (defined by WHO Constitution as “a state of complete physical, mental and social wellbeing, and not merely the absence of disease or infirmity” [27], Preamble]) and rights to other essential determinants of health (such as an adequate standard of living and water and sanitation). With international law framing governments’ legal obligations, human rights law adds moral clarity, political urgency, and legal accountability to the imperative to address FGS [28]. Global health governance institutions have looked to implement human rights as a foundation for global health policy, encompassing healthcare, upstream social determinants of health, and scientific research.

Healthcare is a key component of the right to health. The UN has delineated legal obligations under the right to health to ensure that health services are

- **available**—in sufficient quantity and with equitable geographic distribution;
- **accessible**—both physically and financially, to ensure that all people, including those with disabilities, can receive care equitably;
- **acceptable**—ethically and culturally suitable for users, including marginalized groups and women; and
- of sufficient **quality**—safe, effective, patient centered, timely, and efficient [29].

As has been found for other NTDs [30,31], efforts to advance FGS control and elimination can be hindered when these legal obligations to progressively realize the right to health go unmet. Insufficient availability of trained professionals, diagnostic supplies, and praziquantel can hamper diagnosis and treatment. Inaccessible services, like school-based MDA that overlooks girls not in school [32], can contribute to failures to achieve population-level control targets. Gendered factors, like the perceived permissibility of speaking openly about sexual health with an opposite gender provider when presenting with FGS symptoms, can render services unacceptable to community members, contributing to their disuse. Global schistosomiasis control and elimination efforts that overlook treatment fail to meet obligations regarding quality, particularly for women and girls with long-lasting sequelae, which can undermine community support for programs focused on disrupting transmission. Conversely, crafting control and elimination programs and policies that take these obligations for availability, accessibility, acceptability, and quality into account from the beginning can realize human rights in support of efforts to advance progress toward global targets.

Realizing human rights in addressing FGS also extends beyond healthcare provision. The persistence of schistosomiasis is a direct consequence of the failure to meet human rights obligations to ensure underlying determinants of health, such as the rights to water and sanitation [33]. Recognition of these health-related human rights can inform an emphasis on engaging the water, sanitation, and hygiene sector throughout implementation of the new WHO NTD roadmap. In the case of FGS, there are additional underlying rights that must be considered for affected women and girls. Historically, women’s and girls’ household chores and caregiving responsibilities have been viewed as falling within the private sphere and thus beyond the scope of public policy. Put another way, rights violations that occur within the home were long considered to be beyond the governance of the state, thereby absolving governments of the responsibility to act. In the context of gender-based violence, the UN has found this to be untrue—governments do, in fact, have an obligation to protect rights holders in the home [34]. Further, the UN has determined that household chores and caregiving responsibilities (even when informal and uncompensated) constitute work and thus must be addressed by labor policy [35]. Where schistosomiasis control and elimination programs recognize men’s gendered exposure to infested water (e.g., through fishing) as a key point of intervention, state obligations to safeguard gender equality and to ensure safe and healthy working conditions necessitate that women’s gendered exposures be similarly prioritized. Complementing targeted interventions for those employed in the fishing sector with programs that address use of infested water within the home can build synergy toward elimination goals.

Eliminating FGS as a public health problem will also require scientific research to fill critical knowledge gaps, including those around the management and treatment of long-term sequelae and the development and distribution of new interventions, including vaccine technologies and alternatives to praziquantel [36]. Such an imperative for future research implicates the right to enjoy the benefits of scientific progress in translating research findings into treatment and distribution [37]. This research should center women and girls as priority populations, and efforts will be needed to include women of reproductive age, including pregnant women, in clinical trials to ensure that treatments and vaccines are safe and effective.

In developing these health policies, programs, and practices to eliminate FGS as a public health problem, a rights-based approach to addressing FGS must further be grounded in cross-cutting human rights principles, including:

- **equality and nondiscrimination**: With disproportionate impact on women and girls, the failure to provide safe living and working conditions and the persistence of nonevidence-based exclusion of pregnant and lactating women from treatment reflect discrimination on the basis of sex [38].
- **participation**: Where policymaking around NTDs sometimes includes only nominal participation from women and girls in the development of prevention, treatment, and control programs, policymakers must expand meaningful participation to enable active priority setting with affected communities [39].
- **accountability**: To create pathways for individuals and communities to pursue redress, states must facilitate accountability through human rights impact assessments of health policies and programs; increased health-related human rights advocacy; adequate monitoring and review of structures, processes, and outcomes; and legal enforcement by judicial systems [39].

Consideration of these human rights principles can inform the design of policies and programs that advance both health and human rights. For example, in developing new point-of-care diagnostics for FGS, researchers can conduct user-centered program design with women and girls to determine the kinds of novel diagnostics that would be acceptable. Implementing such rights-based approaches through local, national, and global governance can support efforts to control and eliminate FGS.

## Responding to the human rights imperatives raised by female genital schistosomiasis

Human rights law provides a critical framework for understanding government obligations to address FGS. However, progressively realizing FGS control and elimination will require coordination across multiple sectors and levels of governance to institute policies, programs, and practices that:

- train health professionals and community health workers to recognize genital presentation and symptomatology, and strengthen laboratory diagnostic capabilities to improve detection;
- ensure that control and elimination efforts explicitly address the ways in which gender shapes access to and experience of care, realizing equality under human rights law;
- strengthen the water, sanitation, and hygiene sector to ensure the enjoyment of the right to sufficient, safe, acceptable, accessible, and affordable water, with special attention to the sanitation and hygiene needs of women and girls;
- develop scientific research to fill critical knowledge gaps, including in devising treatment and management strategies for long-term sequelae (such as uterine and tubal masses and cervical lesions) and developing and distributing new vaccine technologies; and
- implement gender-disaggregated indicators through routine collection and reporting of data on FGS, supporting monitoring and review, allowing redress, and facilitating accountability at the national and supranational levels.

Managing and coordinating such efforts will require strong health governance in endemic countries. Domestic FGS policy approaches should be guided by global health models, human rights obligations, and rights-based principles. Implementing effective practices will require broad coalitions of government and civil society stakeholders, including national human rights institutions [40]. Working in partnership with affected communities, with meaningful participation from women and girls, human rights institutions can strengthen ongoing efforts by developing human rights impact assessments and tracking progress on FGS—similar to the human rights oversight mechanisms developed and deployed in responding to obstetric fistula [41]. These rights-based national efforts can help inform future work at the global level to revise the NTD roadmap to strengthen the focus on gender and human rights as a foundation of FGS efforts.

Global governance holds a crucial role in supporting domestic policy and coordinating international assistance and cooperation to realize rights [42]. As many FGS-affected countries are low income, international assistance is needed as countries progressively realize health-related human rights in FGS policies, programs, and practices. WHO can provide technical assistance to guide member states to support the incorporation of human rights into FGS control and elimination efforts. The UN human rights system can facilitate international accountability, with relevant UN treaty bodies providing an institutional basis to monitor and review state progress to realize human rights obligations. For example, many health-related rights provisions fall under the monitoring and review mandate of the UN Committee on Economic, Social, and Cultural Rights, which has previously interpreted state obligations on rights relevant to FGS elimination, including the rights to health [29], water and sanitation [43], nondiscrimination [44], safe working conditions [45], and scientific benefits [37]. Finally, all levels of the global health governance landscape—including donors and researchers—must cooperate to identify opportunities to address FGS across health silos, especially within related areas like HIV/AIDS, cervical cancer, and sexual and reproductive health. Initial collaborative efforts by international organizations, including WHO and UNAIDS, provide a cooperative foundation that should be strengthened, mobilizing human rights to support FGS control and elimination [46].

## Conclusions

Efforts to address FGS must confront the compounding forces of stigma, inequality, and poverty—demanding an intersectional human rights response. Despite affecting millions worldwide—with clear linkages across gender, equity, and rights—FGS remains disconnected from the global SRHR movement. Moving beyond a purely biomedical perspective, human rights obligations offer new avenues to bring moral clarity, political urgency, and legal accountability to this overlooked threat, developing NTD policies, programs, and practices to realize human rights in global health.
